# Association of cardiovascular magnetic resonance-derived circumferential strain parameters with the risk of ventricular arrhythmia and all-cause mortality in patients with prior myocardial infarction and primary prevention implantable cardioverter defibrillator

**DOI:** 10.1186/s12968-019-0536-5

**Published:** 2019-05-16

**Authors:** Elisabeth H. M. Paiman, Alexander F. A. Androulakis, Rahil Shahzad, Qian Tao, Katja Zeppenfeld, Hildo J. Lamb, Rob J. van der Geest

**Affiliations:** 1 Department of Radiology, Leiden University Medical Center, P.O. Box 9600, postal zone C2-S, 2300 RC Leiden, The Netherlands; 2 Department of Cardiology, Leiden University Medical Center, P.O. Box 9600, postal zone C2-S, 2300 RC Leiden, The Netherlands; 3 LKEB, Division of Image Processing, Department of Radiology, Leiden University Medical Center, P.O. Box 9600, postal zone C2-S, 2300 RC Leiden, The Netherlands

**Keywords:** Ventricular arrhythmia, Circumferential strain, Ischemic cardiomyopathy, Magnetic resonance

## Abstract

**Background:**

Impaired left ventricular (LV) contraction and relaxation may further promote adverse remodeling and may increase the risk of ventricular arrhythmia (VA) in ischemic cardiomyopathy. We aimed to examine the association of cardiovascular magnetic resonance (CMR)-derived circumferential strain parameters for LV regional systolic function, LV diastolic function and mechanical dispersion with the risk of VA in patients with prior myocardial infarction and primary prevention implantable cardioverter defibrillator (ICD).

**Methods:**

Patients with an ischemic cardiomyopathy who underwent CMR prior to primary prevention ICD implantation, were retrospectively identified. LV segmental circumferential strain curves were extracted from short-axis cine CMR. For LV regional strain analysis, the extent of moderately and severely impaired strain (percentage of LV segments with strain between − 10% and − 5% and > − 5%, respectively) were calculated. LV diastolic function was quantified by the early and late diastolic strain rate. Mechanical dispersion was defined as the standard deviation in delay time between each strain curve and the patient-specific reference curve. Cox proportional hazard ratios (HR) (95%CI) were calculated to assess the association between LV strain parameters and appropriate ICD therapy.

**Results:**

A total of 121 patients (63 ± 11 years, 84% men, LV ejection fraction (LVEF) 27 ± 9%) were included. During a median (interquartile range) follow-up of 47 (27;69) months, 30 (25%) patients received appropriate ICD therapy. The late diastolic strain rate (HR 1.1 (1.0;1.2) per − 0.25 1/s, *P* = 0.043) and the extent of moderately impaired strain (HR 1.5 (1.0;2.2) per + 10%, *P* = 0.048) but not the extent of severely impaired strain (HR 0.9 (0.6;1.4) per + 10%, *P* = 0.685) were associated with appropriate ICD therapy, independent of LVEF, late gadolinium enhancement (LGE) scar border size and acute revascularization. Mechanical dispersion was not related to appropriate ICD therapy (HR 1.1 (0.8;1.6) per + 25 ms, *P* = 0.464).

**Conclusions:**

In an ischemic cardiomyopathy population referred for primary prevention ICD implantation, the extent of moderately impaired strain and late diastolic strain rate were associated with the risk of appropriate ICD therapy, independent of LVEF, scar border size and acute revascularization. These findings suggest that disturbed LV contraction and relaxation may contribute to an increased risk of VA after myocardial infarction.

**Electronic supplementary material:**

The online version of this article (10.1186/s12968-019-0536-5) contains supplementary material, which is available to authorized users.

## Background

Ventricular arrhythmia (VA) risk stratification in patients with ischemic cardiomyopathy remains challenging. Left ventricular (LV) ejection fraction (LVEF) is currently the major determinant in the selection of post-infarct patients for implantable cardioverter defibrillator (ICD) therapy for the primary prevention of sudden death [1]; however, appropriate ICD therapy is documented in only 35% of the patients [2].

Adverse post-infarct LV remodeling is known to further increase the risk of VA [3, 4]. Disturbances in LV mechanics are recognized as important contributors to progressive ischemic remodeling [5–7]. LV strain is considered a more sensitive measure of contractile function as compared to LVEF [8]. Impairments in both circumferential and longitudinal strain have been found to be predictive of LV remodeling after myocardial infarction [9, 10]. In particular impaired global circumferential strain may be related to ischemic remodeling, as the circumferential function helps to maintain LV structure after severe impairment of the longitudinal function [10, 11]. Also, the LV sphericity index, as a marker of adverse LV remodeling, may be associated with the risk of VA [12, 13]. In recent years, several transthoracic echocardiography studies have shown the potential of LV strain parameters for the prediction of VA [14–17]. However, none of these echocardiography studies have assessed the predictive value of LV strain parameters in comparison with infarct tissue characteristics. Of interest, with the introduction of cardiovascular magnetic resonance (CMR) feature tracking it has become feasible to semi-automatically quantify myocardial circumferential strain based on balanced steady-state free precession (bSSFP) short-axis cine CMR [18, 19]. LV mechanical parameters indicative of adverse remodeling can be derived from standard CMR examinations, in addition to late gadolinium enhancement (LGE) scar characteristics.

Notably, pathologic LV remodeling after myocardial infarction may predispose to VA but also to progressive heart failure [10]. In patients with ischemic cardiomyopathy and primary prevention ICD, non-sudden death due to severe heart failure has been demonstrated to be an important competing risk of sudden death [20]. Previously, a large number of LV segments with systolic dysfunction soon after myocardial infarction has been shown to be an independent predictor of adverse outcome [21, 22]. Possibly, patients with a high extent of severely impaired strain may be at risk of death due to progressive heart failure, whereas those with a high extent of moderately impaired strain may be at specific risk of arrhythmia related death. In this regard, the assessment of LV regional systolic strain may help to distinguish patients with ischemic cardiomyopathy at risk of VA in particular and may add to the identification of individuals who may benefit from ICD therapy.

As well as disturbed LV contractility, impaired LV diastolic function is related to LV remodeling [23]. Recently, an echocardiography study has provided evidence that LV diastolic dysfunction, in particular impairment of the late diastolic tissue velocity, is predictive of VA [16]. Furthermore, as global strain reflects the amplitude but not the timing of the contraction, new indices have been proposed, for example mechanical dispersion [24]. In previous echocardiography studies, mechanical dispersion has been shown to be associated with VA [14, 15, 25]. Prior studies have demonstrated that LV segmental strain [26], but also LV diastolic function [27] and mechanical dispersion [28] can be extracted from bSSFP cine CMR.

The aim of this hypothesis generating study was to assess the association of LV circumferential strain parameters for LV regional systolic function (i.e. extent of moderately and severely impaired strain), LV diastolic function (i.e. early and late diastolic strain rate) and mechanical dispersion, derived from short-axis cine CMR, with appropriate ICD therapy in patients with prior myocardial infarction referred for primary prevention ICD, independent of LVEF and LGE scar border size. Additionally, the relation of LV strain parameters with the competing risk of all-cause mortality without appropriate ICD therapy was examined.

## Methods

### Study population

The study was performed at the Leiden University Medical Center (Leiden, The Netherlands). Consecutive patients with myocardial infarction referred for primary prevention ICD implantation between May 2003 and May 2012 were retrospectively included if a CMR was performed prior to ICD implantation. Part of this population has been previously described [29]. Patients who underwent surgical LV reconstruction within one year after CMR were excluded. For those with late LV repair, follow-up data after surgery were censored. Patients received a dual or single chamber ICD or an ICD combined with cardiac resynchronization therapy (CRT-ICD), according to the guidelines of the European Society of Cardiology (ESC) valid at the time of implantation [30–32]. The assessment of LV function to determine the patient’s eligibility for primary prevention ICD implantation was based on CMR or echocardiography (at another time point). The Dutch Central Committee on Human-related Research (CCMO) allows use of anonymous data without prior approval of an institutional review board provided that the data is acquired for patient care and that the data contains no identifiers that could be traced back to the individual patient. All data used for this study were acquired for clinical treatment, and were stripped of any identifying information. For the present retrospective study, informed consent was waived by the institutional medical research ethics committee.

### Clinical parameters

Clinical baseline characteristics were retrieved from the patients’ medical records. A creatinine serum level ≥ 1.4 mg/dL was considered to indicate renal failure. Presence of a significant stenosis in ≥2 coronary arteries was regarded as multi-vessel disease. Patients with a single myocardial infarction and reperfusion therapy within 24 h from onset of symptoms were categorized as having received acute revascularization.

### CMR data acquisition

Patients were scanned on a 1.5 T Gyoscan ACS-NT/Intera CMR scanner (Philips Healthcare, Best, The Netherlands) using a 5-element cardiac coil. After obtaining scout views, a stack of short-axis slices comprising the complete LV was acquired, using an electrocardiographic triggered bSSFP sequence. Typical imaging parameters were: field of view 400 × 320 mm^2^, matrix 256 × 206, voxel size 1.56 × 1.56 mm^2^, number of slices 12–18, slice thickness 10 mm, slice gap 0 mm, flip angle 35°, echo/repetition time 1.7/3.6 ms, number of phases 30–40. Additionally, LGE imaging in short-axis view, covering the complete LV, was performed approximately 15 min after administration of 0.15 mmol/kg gadolinium diethylenetriamine penta-acetic acid (Magnevist, Bayer Healthcare, Berlin, Germany), using an inversion-recovery 3D turbo-field echo sequence with imaging parameters as previously described [29].

### CMR data analysis

MASS Research Software V2016-EXP (Leiden University Medical Centre, Leiden, The Netherlands) was used for the extraction of the LV circumferential strain curves and the quantification of the LGE scar size. The LV circumferential strain-derived parameters were calculated using MATLAB R2015a (MathWorks, Natick, Massachusetts, USA). Based on a spatiotemporal feature tracking approach, the manually outlined endocardial contours at end-diastole were automatically traced over the entire cardiac cycle [33, 34]. Group-wise image registration was performed to calculate the deformation field, which was used to track the endocardial contours. Basal slices with intersection of the aorta outflow and the lower two apical slices were excluded. Endocardial strain curves were calculated for twelve segments per slice.

LV circumferential strain parameters for global function were global strain, the peak systolic strain rate and the early and late peak diastolic strain rate. Global strain and peak strain rates were defined as the average of peak systolic strain and peak strain rate, respectively, of all segments. Peak systolic strain rate was defined as the minimum strain rate between end-diastole and maximum peak systolic strain. The early and late diastolic strain rate were measured as the maximum strain rate between maximum peak systolic strain and mid-diastole and between mid- and end-diastole, respectively. A comparable approach has previously been applied in tagged and strain-encoded CMR [35]. For curves with peak systolic strain of > − 5% and oscillating curves, the assigned peak systolic, early and late diastolic strain rate was defined as zero, as those segments were assumed to have lost the physiological strain patterns.

To assess LV regional systolic function, the LV was characterized according to the extent of different categories of impaired strain (severely, moderately and mildly impaired strain). Reported normal values for feature tracking-based global circumferential strain are − 23% (− 24.3 to − 21.7%) [36]. As feature tracking-based strain calculation has been recently introduced, no reference values for segmental circumferential strain are available. We presumed that the discriminative ability in segmental peak systolic strain would be at best around 5% based on the acquisition, image analysis and patient population. Therefore, each category of segmental peak systolic strain was spanning a range of 5%. The first category ranging from − 20% to − 15% was regarded as relatively preserved strain, whereas the other categories were assumed to include segments with impaired strain. Accordingly, the following cut-off values were applied: > − 5% (severely impaired strain), − 10% to − 5% (moderately impaired strain) and − 15% to − 10% (mildly impaired strain).

To derive mechanical dispersion, the strain curves were clustered according to the similarity of the motion patterns to constitute a patient-specific reference curve, after exclusion of the curves with peak systolic strain of > − 5% and the oscillating curves. Delay time between the curve of each segment and the reference curve was calculated by cross correlation. Mechanical dispersion was defined as the standard deviation in delay time between each strain curve and the reference curve, as previously proposed [37].

LV sphericity was calculated as the ratio of the LV end-diastolic volume (derived from the short-axis images) to the volume of a sphere with a diameter equal to the LV end-diastolic 4-chamber length, as described previously [12]. LGE scar size was measured according to a previously described semi-automatic scar identification method [29]. The LGE total and border scar zone were defined as myocardium with a signal intensity (SI) > 35% of the maximum SI and with SI ≥35% but < 50% of the maximum SI, respectively. The scar size was quantified in gram, with an estimated myocardial density of 1.05 g/mL. The investigators (EHMP, QT and RJvdG) were blinded to the clinical baseline characteristics and events during the CMR data analysis.

### Follow-up and events

ICD device interrogation was scheduled two months after implantation and every six months thereafter. ICD devices were typically programmed to include three zones: monitor zone (150–188 beats per minute; antitachycardia pacing (ATP) if indicated), fast ventricular tachycardia (VT) zone (188–210 beats per minute; ATP and shock), and ventricular fibrillation (VF) zone (> 210 beats per minute; if available ATP during charging, and shock). Appropriate ICD therapy was defined as ATP or shock subsequent to monomorphic VT or VF. All-cause mortality without appropriate ICD therapy was defined as death without documented appropriate ICD discharge. In case of incomplete follow-up for ICD therapy until death, data were censored after the last ICD device interrogation. The investigator who collected the clinical patient data (AFAA) was blinded to the CMR data.

### Statistical analysis

Statistical analyses were performed using STATA 14.1 (StataCorp LLC, College Station, Texas, USA). The independent-samples Student’s t-test or Fisher's exact test was used to assess the differences in baseline characteristics. Cox proportional hazards regression models were constructed to examine the association of the baseline variables with appropriate ICD therapy and all-cause mortality without appropriate ICD therapy. The Harrell’s C-statistic was calculated to assess the discriminative performance of each baseline variable for appropriate ICD therapy. The proportional hazards assumption was satisfied for all CMR parameters based on the assessment of the Schoenfeld residuals and the time interaction terms. In addition, Kaplan-Meier curves were constructed for the extent of severely and moderately impaired strain, early and late diastolic strain rate and mechanical dispersion. The difference between patients with values below vs. above the observed median in the population in the cumulative incidence of appropriate ICD therapy was assessed using the log-rank test.

Nested Cox regression modeling was performed to test whether the CMR parameters for LV regional strain, LV diastolic function and mechanical function improved the fit of the model for appropriate ICD therapy; first, in comparison to a null model containing LVEF and scar border size; second, in comparison to a null model comprising LVEF, scar border size and the clinical parameter with the best discriminative ability based on the C-statistic in univariable Cox regression analysis. Only one clinical parameter was added to the reference model, as the number of covariables in the model is limited by the number of events in the population. The differences between the extended and the null models were assessed for statistical significance using the χ^2^ likelihood ratio (LR) test. Multi-collinearity was ruled out by calculation of the correlation matrix of the coefficients in the Cox regression model (all correlation coefficients were below 0.75). Additionally, we performed a sensitivity analysis. According to guidelines, LV function to determine the patient’s eligibility for primary prevention ICD implantation should be assessed at least 40 days post myocardial infarction or at least 3 months after revascularization [38]. Therefore, we calculated the Cox hazard ratio for the patients with CMR in the acute/subacute phase and for those with CMR in the chronic stage of myocardial infarction (CMR < or > 40 days after myocardial infarction and < or > 3 months following revascularization, respectively). Intra-observer agreement was evaluated by the intra-class correlation coefficient (ICC) for absolute agreement using a two-way random model based on a random sample of 20 patients from the total population. All statistical tests were two-sided and a *P* value below 0.05 was considered statistically significant.

## Results

### Baseline characteristics

A total of 149 patients with previous myocardial infarction and CMR prior to primary prevention ICD implantation were identified. Eleven patients were excluded due to insufficient image quality (*n* = 3 for short-axis cine and *n* = 8 for LGE CMR) and 17 because of surgical LV reconstruction. The remaining 121 patients (63 ± 11 years; 102 (84%) men) were included. In 77 (64%) and the remaining 44 (36%) patients, ICD indication was based on the 2003 ESC guidelines update (IIa recommendation if LVEF < 30%) and the 2008 ESC guidelines (Ia recommendation if LVEF ≤35% and New York Heart Association (NYHA) Functional Classification ≥II despite optimal medical therapy), respectively [30, 31].

During a median (interquartile range (IQR)) follow-up of 47 (27, 69) months, 30 (25%) received appropriate ICD therapy and 23 (19%) died without having received appropriate ICD therapy. Three patients who died without documented ICD therapy had no complete ICD follow-up until death (last ICD device interrogation was 6–12 months before death). Median (IQR) duration was 22 (0, 168) days between myocardial infarction and CMR acquisition and 34 (9, 125) days between CMR and ICD implantation.

### Clinical parameters

Among patients with as compared to those without appropriate ICD therapy the percentage of acute revascularization was lower. Patients who died without appropriate ICD therapy as compared to those who survived or received appropriate ICD therapy had more often multi-vessel disease, NYHA class III-IV or IV, diabetes mellitus, and used more frequently angiotensin-converting-enzyme (ACE) inhibitors. The clinical baseline characteristics are presented according to appropriate ICD therapy and all-cause mortality without appropriate ICD therapy (Table 1).

**Table 1:** Baseline clinical variables

	No appropriate ICD therapy (*n* = 91)	Appropriate ICD therapy (*n* = 30)	Survived or appropriate ICD therapy (*n* = 98)	Deceased without appropriate ICD therapy (*n* = 23)
Age, years	64 ± 10	63 ± 13	63 ± 11	66 ± 8
Men	74/91 (81%)	28/30 (93%)	81/98 (83%)	21/23 (91%)
Smoking	48/89 (54%)	15/27 (56%)	50/94 (53%)	13/22 (59%)
Hypertension	40/85 (47%)	10/29 (34%)	41/92 (45%)	9/22 (41%)
Hypercholesterolemia	46/76 (61%)	14/24 (58%)	48/80 (60%)	12/20 (60%)
Diabetes mellitus	23/91 (26%)	3/30 (10%)	17/98 (17%)*	9/23 (39%)
Renal failure	16/91 (18%)	10/30 (33%)	18/98 (18%)	8/23 (35%)
Atrial fibrillation	18/91 (20%)	7/30 (23%)	19/98 (19%)	6/23 (26%)
Left bundle branch block	29/91 (32%)	11/30 (37%)	33/98 (34%)	7/23 (30%)
QRS > 120 ms	26/91 (29%)	11/30 (37%)	26/98 (27%)	11/23 (48%)
CRT device	67/91 (74%)	23/30 (77%)	72/98 (73%)	18/23 (78%)
NYHA III-IV	32/91 (35%)	14/30 (47%)	32/98 (33%)†	14/23 (61%)
NYHA IV	4/91 (4%)	1/30 (3%)	1/98 (1%)†	4/23 (17%)
Multi-vessel disease	60/90 (67%)	24/29 (83%)	62/96 (65%)†	22/23 (96%)
Acute revascularization	53/91 (58%)†	8/27 (27%)	51/98 (52%)	10/23 (43%)
Prior CABG	33/91 (36%)	12/30 (40%)	34/98 (35%)	11/23 (48%)
Medication				
Statins	76/90 (84%)	24/30 (80%)	79/98 (81%)	21/22 (95%)
ACE inhibitor	65/90 (72%)	16/30 (53%)	62/98 (63%)*	19/22 (86%)
Aldosterone antagonist	29/90 (32%)	9/30 (30%)	32/98 (33%)	6/22 (27%)
Amiodarone	8/90 (9%)	4/30 (13%)	12/98 (12%)	0/22 (0%)
ARB	15/90 (17%)	6/30 (20%)	19/98 (19%)	2/22 (9%)
Beta blocker	76/90 (84%)	26/30 (87%)	84/98 (86%)	18/22 (82%)
Calcium channel blocker	5/90 (6%)	3/30 (10%)	8/98 (8%)	0/22 (0%)
Any diuretic	54/90 (60%)	21/30 (70%)	61/98 (62%)	14/22 (64%)

Continuous data are expressed as means ± standard deviation and categorical data as numbers (percentages). The presented percentages may not be equal to the percentages of the total number of patients due to missing values. *P < 0.05, †*P* < 0.01, ‡*P* < 0.001 vs. appropriate ICD therapy or all-cause mortality without appropriate ICD therapy. ACE inhibitor: angiotensin-converting-enzyme inhibitor. ARB: angiotensin receptor blocker. CABG: coronary artery bypass graft. CRT: cardiac resynchronization therapy. NYHA: New York Heart Association Functional Classification

### CMR parameters

LVEF (mean ± SD) was 27 ± 9%, total scar size: 49 ± 27 g, scar core size: 30 ± 21 g, scar border size: 20 ± 10 g, global strain: − 13.3 ± 3.9%, peak systolic strain rate: − 0.79 ± 0.27 1/s, extent of severely impaired strain: 16 ± 12%, extent of moderately impaired strain: 25 ± 10%, extent of mildly impaired strain: 23 ± 8%, early diastolic strain rate: 0.76 ± 0.29 1/s, late diastolic strain rate: 0.50 ± 0.23 1/s, mechanical dispersion: 83 ± 24 ms and LV sphericity index: 0.55 ± 0.14. Patients with as compared to those without appropriate ICD therapy had a lower LVEF, higher extent of scar border size, lower global strain, lower peak systolic strain rate, higher extent of severely and moderately impaired strain and a lower early and late diastolic strain rate. In patients who died without appropriate ICD therapy as compared to those who survived or received appropriate ICD therapy, total scar size and scar core size were larger, peak systolic strain rate was lower, the extent of severely impaired strain was larger, late diastolic strain rate was lower and mechanical dispersion was larger. The CMR results are summarized according to appropriate ICD therapy and all-cause mortality without appropriate ICD therapy (Table 2).

**Table 2:** Baseline CMR variables

	No appropriate ICD therapy (*n* = 91)	Appropriate ICD therapy (*n* = 30)	Survived or appropriate ICD therapy (*n* = 98)	Deceased without appropriate ICD therapy (*n* = 23)
LVEF, %	29 ± 9†	23 ± 10	28 ± 9	25 ± 10
Total scar size, g	48 ± 28	55 ± 25	47 ± 24*	60 ± 38
Scar core size, g	29 ± 22	33 ± 19	28 ± 17*	39 ± 31
Scar border size, g	19 ± 9*	23 ± 11	19 ± 10	21 ± 10
LV mass, g	151 ± 35	160 ± 42	152 ± 37	160 ± 35
LV end-diastolic volume, mL	291 ± 104	319 ± 96	290 ± 95	333 ± 128
Global strain, %	−14 ± 4†	−11 ± 3	−14 ± 4	−12 ± 4
Peak systolic strain rate, 1/s	−0.83 ± 0.27†	−0.66 ± 0.20	−0.81 ± 0.27*	−0.68 ± 0.24
Extent of impaired strain, %				
Severely (<−5%)	15 ± 11*	20 ± 14	15 ± 11*	21 ± 14
Moderately (−5, −10%)	23 ± 10‡	30 ± 10	24 ± 11	26 ± 9
Mildly (−10, −15%)	23 ± 8	22 ± 7	23 ± 8	22 ± 7
Early diastolic strain rate, 1/s	0.79 ± 0.30*	0.64 ± 0.25	0.78 ± 0.29	0.68 ± 0.26
Late diastolic strain rate, 1/s	0.52 ± 0.24*	0.42 ± 0.20	0.52 ± 0.23*	0.40 ± 0.23
Sphericity index	0.54 ± 0.13	0.57 ± 0.17	0.54 ± 0.14	0.58 ± 0.17
Mechanical dispersion, ms	82 ± 24	83 ± 25	80 ± 24*	92 ± 21

Means ± standard deviations. *P < 0.05, †*P* < 0.01, ‡*P* < 0.001 vs. appropriate ICD therapy or all-cause mortality without appropriate ICD therapy. Missing values for sphericity index: *n* = 2 (no appropriate ICD therapy); n = 1 (appropriate ICD therapy); n = 2 survived or appropriate ICD therapy; *n* = 1 deceased without appropriate ICD therapy. LV: left ventricle. LVEF: left ventricular ejection fraction. Extent of impaired strain: percentage of LV segments with strain > − 15%

All circumferential strain-derived measures were highly reproducible. The ICC (95%CI) was 0.96 (0.90, 0.98) for severely impaired strain, 0.93 (0.84, 0.97) for moderately impaired strain, 0.98 (0.95, 0.99) for early diastolic strain rate, 0.97 (0.93, 0.99) for late diastolic strain rate, and 0.96 (0.90, 0.98) for mechanical dispersion.

### Appropriate ICD therapy

**Fig. 1:**
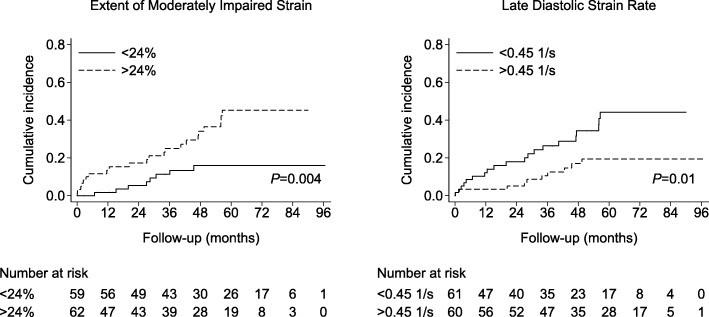
Kaplan-Meier curves for the cumulative incidence of appropriate implantable cardioverter defibrillator (ICD) therapy, with the observed median as the cut-off. *P* values for the log-rank test are shown

The risk of appropriate ICD therapy was higher for patients without acute revascularization, for those with multi-vessel disease, renal failure, a relatively lower LVEF, larger total scar size, larger scar border size, lower global strain, lower peak systolic strain rate, a higher extent of severely and moderately impaired strain, a lower early and late diastolic strain rate. In contrast, mechanical dispersion and the LV sphericity index were not associated with the risk of appropriate ICD therapy (Tables 3 and 4). Furthermore, the incidence of appropriate ICD therapy was significantly higher for patients with a relatively high extent of moderately impaired strain or a relatively low early and late diastolic strain rate (log-rank test *P* = 0.004, *P* = 0.01 and *P* = 0.01, respectively) (Fig. 1). In contrast, no differences in the cumulative incidence curves of appropriate ICD therapy were observed for the extent of severely impaired strain or mechanical dispersion (log-rank test *P* = 0.215 and *P* = 0.813, respectively).

**Table 3:** Unadjusted Cox hazard ratio for the clinical parameters

	Appropriate ICD therapy (30/121)			All-cause mortality without appropriate ICD therapy (23/121)		
	Cox HR (95%CI)	*P* value	Harrell’s C-statistic	Cox HR (95%CI)	*P* value	Harrell’s C-statistic
Age, per + 1 year	1.0 (1.0, 1.0)	0.785	0.49	1.0 (1.0, 1.1)	0.353	0.50
Men	3.5 (0.8, 15)	0.086	0.57	2.8 (0.7, 12)	0.164	0.57
Smoking	1.1 (0.5, 2.3)	0.882	0.51	1.4 (0.6, 3.4)	0.449	0.55
Hypertension	0.6 (0.3, 1.3)	0.193	0.54	0.7 (0.3, 1.8)	0.543	0.53
Hypercholesterolemia	1.0 (0.4, 2.2)	0.958	0.50	1.0 (0.4, 2.5)	0.941	0.50
Diabetes mellitus	0.4 (0.1, 1.4)	0.154	0.56	2.2 (1.0, 5.2)	0.062	0.59
Renal failure	2.3 (1.1, 5.0)	0.028	0.58	2.5 (1.1, 6.0)	0.038	0.59
Atrial fibrillation	1.2 (0.5, 2.9)	0.636	0.53	1.5 (0.6, 3.7)	0.426	0.53
Left bundle branch block	1.1 (0.5, 2.3)	0.789	0.52	0.8 (0.3, 2.0)	0.689	0.52
QRS > 120 ms	1.5 (0.7, 3.3)	0.249	0.56	2.3 (1.0, 5.2)	0.051	0.58
CRT device	1.1 (0.5, 2.6)	0.825	0.51	1.3 (0.5, 3.3)	0.661	0.49
NYHA III-IV	1.8 (0.9, 3.7)	0.113	0.58	3.0 (1.3, 6.9)	0.012	0.62
NYHA IV	1.3 (0.2, 9.7)	0.785	0.51	5.6 (1.7, 18)	0.004	0.56
Acute revascularization	0.3 (0.1, 0.7)	0.003	0.65	0.6 (0.2, 1.3)	0.185	0.54
Multi-vessel disease	2.8 (1.0, 7.2)	0.040	0.61	12 (1.7, 92)	0.014	0.65
Prior CABG	1.2 (0.6, 2.5)	0.628	0.53	1.6 (0.7, 3.7)	0.251	0.53
Medication						
Statins	0.8 (0.3, 2.0)	0.621	0.62	4.1 (0.5, 30)	0.170	0.56
ACE inhibitor	0.6 (0.3, 1.2)	0.116	0.57	2.9 (0.9, 10)	0.081	0.59
Aldosterone antagonist	0.9 (0.4, 1.9)	0.729	0.50	0.8 (0.3, 2.1)	0.678	0.55
Amiodarone	1.3 (0.4, 3.6)	0.666	0.52	–		
ARB	1.1 (0.4, 2.6)	0.867	0.50	0.5 (0.1, 2.0)	0.308	0.54
Beta blocker	1.1 (0.4, 3.1)	0.903	0.49	0.7 (0.2, 2.1)	0.552	0.52
Calcium channel blocker	1.5 (0.5, 5.1)	0.476	0.52	–		0.53
Any diuretic	1.3 (0.6, 2.9)	0.456	0.53	1.1 (0.4, 2.6)	0.882	0.47

Abbreviations as in Table 1. The discriminative performance of each parameter for appropriate ICD therapy or all-cause mortality without appropriate ICD therapy is indicated by the Harrell’s C-statistic (no, good, excellent and perfect discriminative ability is indicated by a C-statistic of 0.5, > 0.7, > 0.8 and 1, respectively). Note: none of the patients who died during follow-up without having received appropriate ICD therapy used amiodarone or calcium channel blockers

**Table 4:** Unadjusted Cox hazard ratio for the CMR parameters

	Appropriate ICD therapy (30/121)			All-cause mortality without appropriate ICD therapy (23/121)		
	Cox HR (95%CI)	*P* value	Harrell’s C-statistic	Cox HR (95%CI)	*P* value	Harrell’s C-statistic
LVEF, per −10%	2.1 (1.4, 3.2)	0.001	0.70	1.5 (0.9, 2.4)	0.084	0.60
Total scar size, per 10 g	1.1 (1.0, 1.3)	0.030	0.65	1.2 (1.0, 1.3)	0.009	0.63
Scar core size, per 10 g	1.1 (1.0, 1.3)	0.115	0.60	1.2 (1.1, 1.4)	0.007	0.61
Scar border size, per 10 g	1.6 (1.2, 2.1)	0.009	0.65	1.4 (0.9, 2.0)	0.122	0.62
Global strain, per + 5%	2.9 (1.6, 5.2)	< 0.001	0.69	2.1 (1.1, 4.0)	0.020	0.64
Peak systolic strain rate, per + 0.25 1/s	2.3 (1.4, 3.7)	0.001	0.68	2.0 (1.2, 3.3)	0.011	0.66
Extent of impaired strain, %						
Severely (<−5%)	1.5 (1.1, 1.9)	0.005	0.64	1.5 (1.1, 2.1)	0.005	0.66
Moderately (−5, −10%)	1.9 (1.4, 2.5)	< 0.001	0.71	1.4 (0.9, 2.0)	0.099	0.63
Mildly (−10, −15%)	0.7 (0.4, 1.2)	0.203	0.59	0.8 (0.4, 1.4)	0.359	0.55
Early diastolic strain rate, per −0.25 1/s	1.1 (1.0, 1.2)	0.005	0.66	1.1 (1.0, 1.1)	0.066	0.61
Late diastolic strain rate, per − 0.25 1/s	1.1 (1.0, 1.2)	0.008	0.66	1.1 (1.0, 1.3)	0.008	0.71
Mechanical dispersion, per + 25 ms	1.1 (0.8, 1.6)	0.464	0.55	1.6 (1.1, 2.3)	0.014	0.66
Sphericity index, per + 0.1	1.2 (0.9, 1.5)	0.271	0.53	1.2 (0.9, 1.6)	0.296	0.58

Abbreviations as in Table 2

**Fig. 2:**
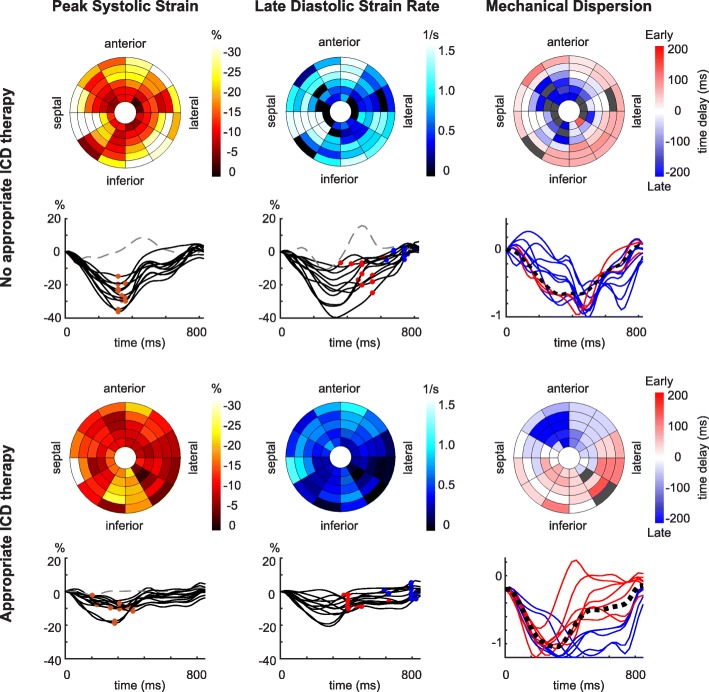
Example of left ventricular (LV) circumferential strain in a patient without and with appropriate ICD therapy. LV bullseye representation of peak systolic strain, late diastolic strain rate and mechanical dispersion and LV segmental strain curves per slice with LV segmental peak systolic strain (orange dots), early diastolic strain rate (red dots), late diastolic strain rate (blue dots) and normalized curves with the patient-specific reference curve (black dotted lines). In the LV bullseye for mechanical dispersion, LV segments with early and late contraction patterns are shown in red and blue, respectively. (Upper panel) 71-year-old woman without appropriate ICD therapy (LV ejection fraction (LVEF) 30%). (Lower panel) 71-year-old man with appropriate ICD therapy at 40 months after ICD implantation (LVEF 26%). In the presented patient with appropriate ICD therapy, the extent of moderately impaired strain (percentage of LV segments with peak systolic strain between − 5% and − 10%) is relatively large, the early and late diastolic strain rate are low, whereas mechanical dispersion is comparable in the presented cases

On multivariable analysis, the extent of moderately impaired strain and late diastolic strain rate were associated with the risk of appropriate ICD therapy, independent of LVEF, scar border size and acute revascularization, and both parameters significantly improved the fit of the model for the risk of appropriate ICD therapy as compared LVEF and scar border zone (C-statistic increased from 0.71 to 0.73 (LR test *P* = 0.041) and 0.73 (LR test *P* = 0.034), respectively) (Table 5). When acute revascularization was additionally included in the reference model for the risk of appropriate ICD therapy, late diastolic strain rate remained to add incremental benefit (C-statistic increased from 0.73 to 0.75, LR test *P* = 0.033), whereas the extent of moderately impaired strain tended to have additive value for the fit of the model (C-statistic increased from 0.73 to 0.76, LR test *P* = 0.056) (Table 5). An example of a patient with appropriate ICD therapy with a relatively large extent of moderately impaired strain and relatively low late diastolic strain rate is provided (Fig. 2).

**Table 5:** Multivariable Cox regression model for appropriate ICD therapy

	Cox HR		Harrell’s	LR test	
	(95%CI)	*P* value	C-statistic	χ^2^	*P* value
LVEF, scar border size			0.71	13.12	Reference
Added to null model:					
Extent of impaired strain, per + 10%					
Severely (<−5%)	1.0 (0.6, 1.5)	0.844	0.71	13.16	0.844
Moderately (−5, −10%)	1.5 (1.0, 2.2)	0.034	0.73	17.30	0.041
Mildly (−10, − 15%)	0.8 (0.5, 1.4)	0.487	0.71	13.62	0.482
Early diastolic strain rate, per − 0.25 1/s	1.1 (1.0, 1.1)	0.179	0.71	15.06	0.164
Late diastolic strain rate, per −0.25 1/s	1.1 (1.0, 1.2)	0.044	0.73	17.64	0.034
Mechanical dispersion, per + 25 ms	1.0 (0.7, 1.5)	0.815	0.71	13.18	0.812
LVEF, scar border size, acute revascularization			0.73	17.65	Reference
Added to null model:					
Extent of impaired strain, per + 10%					
Severely (<−5%)	0.9 (0.6, 1.4)	0.685	0.73	17.82	0.685
Moderately (−5, −10%)	1.5 (1.0, 2.2)	0.048	0.76	21.30	0.056
Mildly (−10, −15%)	0.8 (0.5, 1.3)	0.403	0.74	18.38	0.394
Early diastolic strain rate, per −0.25 1/s	1.0 (1.0, 1.1)	0.355	0.73	18.54	0.345
Late diastolic strain rate, per −0.25 1/s	1.1 (1.0, 1.2)	0.043	0.75	22.19	0.033
Mechanical dispersion, per + 25 ms	1.1 (0.8, 1.7)	0.490	0.74	18.12	0.495

Abbreviations as in Table 2. The incremental value of each LV strain parameter for the fit of the Cox regression model for the risk of appropriate ICD therapy as compared to the null model was assessed using the likelihood ratio (LR) chi-square statistic (χ^2^)

### All-cause mortality without appropriate ICD therapy

The clinical parameters multi-vessel disease, NYHA class III-IV or IV and renal failure were associated with an increased risk of death without having received appropriate ICD therapy. Regarding the CMR parameters, a larger total scar size, larger scar core size, lower global strain, lower peak systolic strain rate, higher extent of severely impaired strain, lower late diastolic strain rate and higher mechanical dispersion were related to an increased risk of all-cause mortality without appropriate ICD therapy (Tables 3 and 4). The LV sphericity index was not associated with the risk of all-cause mortality without having received appropriate ICD therapy.

### Sensitivity analysis

CMR was acquired in the acute/subacute phase vs. chronic stage in 72 and 49 patients, respectively. In the acute/subacute and chronic subgroup, respectively, 18/72 (25%) and 12/49 (24%) received appropriate ICD therapy and 9/72 (13%) and 14/49 (29%) died without having received appropriate ICD therapy. In the acute/subacute vs. the chronic subgroup, the risk of appropriate ICD therapy was comparable in relation to the scar border size (HR 1.6 (0.9, 2.6) and 1.6 (1.0, 2.5) per + 10 g, respectively), the extent of moderately impaired strain (HR 1.9 (1.3, 2.7) and 1.8 (1.1, 3.1) per + 10%, respectively) and late diastolic strain rate (HR 1.1 (1.0, 1.2) and 1.1 (1.0, 1.3) per − 0.25 1/s, respectively). In contrast, the risk of appropriate ICD therapy in relation to LVEF was lower in the acute/subacute compared to the chronic subgroup (HR 1.9 (1.1, 3.4) per − 10% vs. 3.1 (1.4, 6.8) per − 10%) (Additional file 1: Table S1).

## Discussion

In this retrospective study of ischemic cardiomyopathy patients undergoing CMR prior to primary prevention ICD implantation, CMR-derived circumferential strain analysis showed that the extent of moderately impaired strain and late diastolic strain rate were associated with the risk of appropriate ICD therapy, independent of LVEF, LGE scar border size and acute revascularization. There was no relation between mechanical dispersion and the risk of appropriate ICD therapy.

### LV segmental strain

Of interest, the extent of moderately impaired strain was specifically related to an increased risk of VA, whereas the extent of severely impaired strain was associated with the risk of both appropriate ICD therapy and all-cause mortality without appropriate ICD therapy. Therefore, the assessment of the extent of moderately impaired strain in particular may be helpful for sudden death risk stratification. Circumferential contractile performance is considered essential for maintaining LV shape and restraining LV dilation [10]. In this regard, the extent of impaired strain may be associated with appropriate ICD therapy, as it may be indicative of an increased susceptibility to ongoing adverse remodeling after myocardial infarction which has been shown to correlate with electrical instability and an increased risk of sudden death [4]. Previously, LV sphericity has been proposed as a marker of adverse LV remodeling and has been shown to be associated with appropriate ICD therapy in patients with ischemic and non-ischemic cardiomyopathy [12, 13]. Notably, in our study population selectively including patients with an ischemic cardiomyopathy, LV mechanical parameters and scar characteristics rather than LV structural indices such as LV sphericity were related to the risk of VA.

Several echocardiography studies have previously addressed LV regional function in relation to adverse outcome in ischemic cardiomyopathy. The regional extent of systolic dysfunction after myocardial infarction has been shown to be independently associated with heart failure and mortality [22, 39]. Also, an independent relation between impaired regional strain in the scar border zone and appropriate ICD therapy has been demonstrated [40]. Most research on CMR-derived LV regional strain has been limited to studies on the predictive value of LV segmental strain for persistent contractile dysfunction shortly after myocardial infarction [41, 42]. To our knowledge, this is the first CMR study in which the association between LV segmental strain and the risk of VA in ischemic cardiomyopathy has been examined.

### LV diastolic function

Late but not early diastolic function was independently associated with appropriate ICD therapy and late but not early diastolic function was associated with all-cause mortality without appropriate ICD therapy. As well as systolic abnormalities, diastolic dysfunction, due to the resulting elevated LV filling pressure and the progressive LV enlargement, may contribute to an increased risk of sudden and non-sudden death [23, 43]. It has to be pointed out that early and late diastolic function reflect different relaxation processes. Whereas early diastolic function is an active, energy requiring process, which is therefore highly susceptible to ischemia, the late diastolic function parameters are predominantly dependent on the passive LV stiffness and left atrial function [44]. Late diastolic function parameters are considered to deteriorate when left atrial function fails to compensate for the progressive increase in the passive LV stiffness [16]. In this regard, in a population with severely depressed LVEF late diastolic function in particular may be indicative of adverse LV remodeling, which may further increase the risk of sudden and non-sudden death.

Our observations are consistent with previous echocardiography studies, in which late but not early diastolic function was associated with adverse cardiac outcome including VA, cardiac mortality and/or heart failure [16, 45]. Our study adds to the current, limited evidence that LV diastolic function may have potential for VA risk stratification in patients with myocardial infarction and reduced LVEF.

### LV mechanical dispersion

In our study population with relatively depressed LVEF, mechanical dispersion was not associated with appropriate ICD therapy. Some echocardiography studies have shown a strong and independent relation between mechanical dispersion and VA [14, 15, 25], whereas others did not find such association [16, 17]. The observed association between mechanical dispersion and death without appropriate ICD therapy is in keeping with other studies, which reported a relation of mechanical dispersion with heart failure and mortality [28, 46]. We speculate that, depending on the cohort characteristics, mechanical dispersion may be a risk stratifier for either sudden or non-sudden death.

### LGE scar

We found that the extent of moderately impaired strain and late diastolic strain rate provided incremental benefit for VA risk stratification above LVEF and LGE scar border size. It remains debated which LGE scar characteristics are most predictive of sudden death. Although several studies have demonstrated that LGE scar border size in particular is indicative of an increased VA susceptibility [29, 47], others observed no association between the border size and the risk of VA [48, 49] or reported a comparable association for border and total scar size [50]. In this regard, CMR-derived LV circumferential strain parameters may or may not have additive value above LGE scar for VA risk stratification, if other approaches for the quantification of infarct tissue heterogeneity would have been applied.

### Limitations

Because of the retrospective design, no conclusions on causality can be drawn. We used CMR as this enabled the assessment of the incremental value of LV strain parameters above LGE scar characteristics for VA risk stratification. Whereas the high number of extracted strain curves in CMR is a plus, disadvantage of CMR as compared to echocardiography is the inferior temporal resolution when analyzing LV diastolic function and mechanical dispersion. Also, our study was limited by the arbitrary definition of the strain categorization of the LV segments and prospective studies are required for validation. Furthermore, the sample size was not sufficient for more detailed subgroup analyses, for example according to the type of arrhythmic event, although our study population was relatively large for a single center cohort.

In our retrospective study, the CMR examinations were acquired in the acute/subacute phase as well as in the chronic stage of myocardial infarction. Importantly, the associations of the scar border size, the extent of moderately impaired strain and late diastolic strain rate with the risk of appropriate ICD therapy were comparable for the subgroups with CMR in the acute/subacute phase and in the chronic stage of myocardial infarction. In contrast, our results suggested that the predictive value of LVEF for appropriate ICD therapy was substantially higher when LVEF was assessed in patients with chronic compared to acute/subacute myocardial infarction. Therefore, prospective studies with CMR at 40 days after myocardial infarction or 3 months after revascularization are needed to confirm the additive value of the extent of moderately impaired strain and late diastolic strain rate for VA risk stratification beyond LVEF.

### Implications

Our findings suggest that both disturbed LV contraction and relaxation increase the risk of VA, which may contribute to a better understanding of the complex pathophysiology of VA in ischemic cardiomyopathy. The extent of impaired LV segmental strain has previously been assessed in relation to adverse outcome including heart failure and mortality [21, 22, 39]. We showed that LV regional strain is also related to the risk of appropriate ICD therapy. Furthermore, our results confirm previous findings that LV diastolic function can be helpful in VA risk stratification and add to the existing evidence that LV diastolic function provides incremental benefit above LGE scar [16].

Patients with appropriate ICD therapy were slightly better identified after assessment of the extent of moderately impaired strain or late diastolic strain rate in addition to LV global function and LGE scar. Our findings indicate that VA risk stratification in ischemic cardiomyopathy can be improved by the evaluation of additional imaging parameters derived from standard clinical CMR examinations. For clinical implementation, our model including LVEF, the scar border size, regional strain and diastolic function, might be extended by other imaging parameters, for example novel scar characteristics, which together may further increase the discriminative performance for appropriate ICD therapy.

## Conclusions

In patients with prior myocardial infarction being considered for primary prevention ICD, the extent of moderately impaired strain and late diastolic strain rate are associated with appropriate ICD therapy, independent of LVEF, LGE scar border size and acute revascularization. In contrast, mechanical dispersion shows no relation with appropriate ICD therapy. Notably, the extent of moderately impaired strain is specifically associated with appropriate ICD therapy, whereas the extent of severely impaired strain is also related to death without having received appropriate ICD therapy. Furthermore, deterioration of late diastolic function in particular may be indicative of adverse LV remodeling in patients with severe LV dysfunction, which may explain the observed association of late rather than early diastolic function with an increased risk of appropriate ICD therapy. This work can be seen as a hypothesis generating study, which may help to elucidate which mechanical parameters are predictive of an increased risk of VA in addition to established functional and scar-related imaging markers. In this study, no longitudinal imaging data was available, which would have provided more insight into the role LV remodeling in LV arrhythmogenesis. Therefore, whether the increased VA vulnerability in association with disturbed LV contraction and relaxation is related to late adverse remodeling needs to be assessed in further research.

## Additional file

Additional file 1: **Table S1.** Unadjusted Cox hazard ratio for the CMR parameters in the acute/subacute phase vs. the chronic stage. (DOCX 19 kb)

## Abbreviations

ACE: Angiotensin-converting-enzyme
ARB: Angiotensin receptor blocker
ATP: Antitachycardia pacing
bSSFP: Balanced steady-state free precession
CABG: Coronary artery bypass graft
CMR: Cardiovascular magnetic resonance
CRT: Cardiac resynchronization therapy
ESC: European Society of Cardiology
HR: Hazard ratio
ICC: Intra-class correlation coefficient
ICD: Implantable cardioverter defibrillator
IQR: Interquartile range
LGE: Late gadolinium enhancement
LR: Likelihood ratio
LV: Left ventricle/left ventricular
LVEF: Left ventricular ejection fraction
NYHA: New York Heart Association Functional Classification
SI: Signal intensity
VA: Ventricular arrhythmia
VF: Ventricular fibrillation
VT: Ventricular tachycardia

## References

[1] Brignole M, Auricchio A, Baron-Esquivias G, Bordachar P, Boriani G, Breithardt OA (2013). 2013 ESC guidelines on cardiac pacing and cardiac resynchronization therapy: the task force on cardiac pacing and resynchronization therapy of the European Society of Cardiology (ESC). Developed in collaboration with the European Heart Rhythm Association (EHRA). Eur Heart J.

[2] Moss AJ, Greenberg H, Case RB, Zareba W, Hall WJ, Brown MW (2004). Long-term clinical course of patients after termination of ventricular tachyarrhythmia by an implanted defibrillator. Circulation..

[3] St John Sutton M, Lee D, Rouleau JL, Goldman S, Plappert T, Braunwald E (2003). Left ventricular remodeling and ventricular arrhythmias after myocardial infarction. Circulation..

[4] Gaudron P, Kugler I, Hu K, Bauer W, Eilles C, Ertl G (2001). Time course of cardiac structural, functional and electrical changes in asymptomatic patients after myocardial infarction: their inter-relation and prognostic impact. J Am Coll Cardiol.

[5] D'Elia N, D'Hooge J, Marwick TH (2015). Association between myocardial mechanics and ischemic LV remodeling. JACC Cardiovasc Imaging.

[6] Mollema SA, Liem SS, Suffoletto MS, Bleeker GB, van der Hoeven BL, van de Veire NR (2007). Left ventricular dyssynchrony acutely after myocardial infarction predicts left ventricular remodeling. J Am Coll Cardiol.

[7] Carluccio E, Biagioli P, Alunni G, Murrone A, Giombolini C, Ragni T (2006). Patients with hibernating myocardium show altered left ventricular volumes and shape, which revert after revascularization: evidence that dyssynergy might directly induce cardiac remodeling. J Am Coll Cardiol.

[8] Mordi I, Bezerra H, Carrick D, Tzemos N (2015). The combined incremental prognostic value of LVEF, late gadolinium enhancement, and global circumferential strain assessed by CMR. JACC Cardiovasc Imaging.

[9] Joyce E, Hoogslag GE, Leong DP, Debonnaire P, Katsanos S, Boden H (2014). Association between left ventricular global longitudinal strain and adverse left ventricular dilatation after ST-segment-elevation myocardial infarction. Circ Cardiovasc Imaging.

[10] Hung CL, Verma A, Uno H, Shin SH, Bourgoun M, Hassanein AH (2010). Longitudinal and circumferential strain rate, left ventricular remodeling, and prognosis after myocardial infarction. J Am Coll Cardiol.

[11] Wang J, Khoury DS, Yue Y, Torre-Amione G, Nagueh SF (2008). Preserved left ventricular twist and circumferential deformation, but depressed longitudinal and radial deformation in patients with diastolic heart failure. Eur Heart J.

[12] Nakamori S, Ismail H, Ngo LH, Manning WJ, Nezafat R (2017). Left ventricular geometry predicts ventricular tachyarrhythmia in patients with left ventricular systolic dysfunction: a comprehensive cardiovascular magnetic resonance study. J Cardiovasc Magn Reson.

[13] Levine YC, Matos J, Rosenberg MA, Manning WJ, Josephson ME, Buxton AE (2016). Left ventricular sphericity independently predicts appropriate implantable cardioverter-defibrillator therapy. Heart Rhythm.

[14] Ersboll M, Valeur N, Andersen MJ, Mogensen UM, Vinther M, Svendsen JH (2013). Early echocardiographic deformation analysis for the prediction of sudden cardiac death and life-threatening arrhythmias after myocardial infarction. JACC Cardiovasc Imaging.

[15] Haugaa KH, Grenne BL, Eek CH, Ersboll M, Valeur N, Svendsen JH (2013). Strain echocardiography improves risk prediction of ventricular arrhythmias after myocardial infarction. JACC Cardiovasc Imaging.

[16] Biering-Sorensen T, Olsen FJ, Storm K, Fritz-Hansen T, Olsen NT, Jons C (2016). Prognostic value of tissue Doppler imaging for predicting ventricular arrhythmias and cardiovascular mortality in ischaemic cardiomyopathy. Eur Heart J Cardiovasc Imaging.

[17] Biering-Sorensen T, Knappe D, Pouleur AC, Claggett B, Wang PJ, Moss AJ, et al. Regional longitudinal deformation improves prediction of ventricular Tachyarrhythmias in patients with heart failure with reduced ejection fraction: a MADIT-CRT substudy (multicenter automatic defibrillator implantation trialcardiac resynchronization therapy). Circ Cardiovasc Imaging. 2017;10(1):e005096. 10.1161/CIRCIMAGING.116.005096 28003221

[18] Schuster A, Hor KN, Kowallick JT, Beerbaum P, Kutty S (2016). Cardiovascular magnetic resonance myocardial feature tracking: concepts and clinical applications. Circ Cardiovasc Imaging..

[19] Hor KN, Baumann R, Pedrizzetti G, Tonti G, Gottliebson WM, Taylor M, et al. Magnetic resonance derived myocardial strain assessment using feature tracking. J Vis Exp. 2011;48. 10.3791/2356 PMC3074463 21372778

[20] Friedman DJ, Al-Khatib SM, Zeitler EP, Han J, Bardy GH, Poole JE (2017). New York heart association class and the survival benefit from primary prevention implantable cardioverter defibrillators: a pooled analysis of 4 randomized controlled trials. Am Heart J.

[21] Bodi V, Sanchis J, Nunez J, Mainar L, Lopez-Lereu MP, Monmeneu JV (2009). Prognostic value of a comprehensive cardiac magnetic resonance assessment soon after a first ST-segment elevation myocardial infarction. JACC Cardiovasc Imaging.

[22] Wang N, Hung CL, Shin SH, Claggett B, Skali H, Thune JJ (2016). Regional cardiac dysfunction and outcome in patients with left ventricular dysfunction, heart failure, or both after myocardial infarction. Eur Heart J.

[23] Temporelli PL, Giannuzzi P, Nicolosi GL, Latini R, Franzosi MG, Gentile F (2004). Doppler-derived mitral deceleration time as a strong prognostic marker of left ventricular remodeling and survival after acute myocardial infarction: results of the GISSI-3 echo substudy. J Am Coll Cardiol.

[24] Haugaa KH, Amlie JP, Berge KE, Leren TP, Smiseth OA, Edvardsen T (2010). Transmural differences in myocardial contraction in long-QT syndrome: mechanical consequences of ion channel dysfunction. Circulation..

[25] Matsuzoe H, Tanaka H, Matsumoto K, Toki H, Shimoura H, Ooka J (2016). Left ventricular dyssynergy and dispersion as determinant factors of fatal ventricular arrhythmias in patients with mildly reduced ejection fraction. Eur Heart J Cardiovasc Imaging.

[26] Khan JN, Singh A, Nazir SA, Kanagala P, Gershlick AH, McCann GP (2015). Comparison of cardiovascular magnetic resonance feature tracking and tagging for the assessment of left ventricular systolic strain in acute myocardial infarction. Eur J Radiol.

[27] Moody WE, Taylor RJ, Edwards NC, Chue CD, Umar F, Taylor TJ (2015). Comparison of magnetic resonance feature tracking for systolic and diastolic strain and strain rate calculation with spatial modulation of magnetization imaging analysis. J Magn Reson Imaging.

[28] Muser D, Tioni C, Shah R, Selvanayagam JB, Nucifora G (2017). Prevalence, correlates, and prognostic relevance of myocardial mechanical dispersion as assessed by feature-tracking cardiac magnetic resonance after a first ST-segment elevation myocardial infarction. Am J Cardiol.

[29] Roes SD, Borleffs CJ, van der Geest RJ, Westenberg JJ, Marsan NA, Kaandorp TA (2009). Infarct tissue heterogeneity assessed with contrast-enhanced MRI predicts spontaneous ventricular arrhythmia in patients with ischemic cardiomyopathy and implantable cardioverter-defibrillator. Circ Cardiovasc Imaging.

[30] Priori SG, Aliot E, Blomstrom-Lundqvist C, Bossaert L, Breithardt G, Brugada P (2003). Update of the guidelines on sudden cardiac death of the European Society of Cardiology. Eur Heart J.

[31] Dickstein K, Cohen-Solal A, Filippatos G, McMurray JJ, Ponikowski P, Poole-Wilson PA (2008). ESC guidelines for the diagnosis and treatment of acute and chronic heart failure 2008: the task force for the diagnosis and treatment of acute and chronic heart failure 2008 of the European Society of Cardiology. Developed in collaboration with the heart failure association of the ESC (HFA) and endorsed by the European Society of Intensive Care Medicine (ESICM). Eur J Heart Fail.

[32] Dickstein K, Vardas PE, Auricchio A, Daubert JC, Linde C, McMurray J (2010). 2010 Focused Update of ESC Guidelines on device therapy in heart failure: an update of the 2008 ESC Guidelines for the diagnosis and treatment of acute and chronic heart failure and the 2007 ESC guidelines for cardiac and resynchronization therapy. Developed with the special contribution of the Heart Failure Association and the European Heart Rhythm Association. Eur Heart J.

[33] Metz CT, Klein S, Schaap M, van Walsum T, Niessen WJ (2011). Nonrigid registration of dynamic medical imaging data using nD + t B-splines and a groupwise optimization approach. Med Image Anal.

[34] Tsadok Y, Friedman Z, Haluska BA, Hoffmann R, Adam D (2016). Myocardial strain assessment by cine cardiac magnetic resonance imaging using non-rigid registration. Magn Reson Imaging.

[35] Neizel M, Lossnitzer D, Korosoglou G, Schaufele T, Lewien A, Steen H (2009). Strain-encoded (SENC) magnetic resonance imaging to evaluate regional heterogeneity of myocardial strain in healthy volunteers: comparison with conventional tagging. J Magn Reson Imaging.

[36] Vo Ha Q., Marwick Thomas H., Negishi Kazuaki (2018). MRI-Derived Myocardial Strain Measures in Normal Subjects. JACC: Cardiovascular Imaging.

[37] Suever JD, Fornwalt BK, Neuman LR, Delfino JG, Lloyd MS, Oshinski JN (2014). Method to create regional mechanical dyssynchrony maps from short-axis cine steady-state free-precession images. J Magn Reson Imaging.

[38] Al-Khatib SM, Stevenson WG, Ackerman MJ, Bryant WJ, Callans DJ, Curtis AB, et al. 2017 AHA/ACC/HRS guideline for Management of Patients with Ventricular Arrhythmias and the prevention of sudden cardiac death: a report of the American College of Cardiology/American Heart Association task force on clinical practice guidelines and the Heart Rhythm Society. J Am Coll Cardiol. 2018;72(14):1677-49. 10.1016/j.jacc.2017.10.053 29097294

[39] Ersboll M, Valeur N, Mogensen UM, Andersen MJ, Moller JE, Hassager C (2012). Relationship between left ventricular longitudinal deformation and clinical heart failure during admission for acute myocardial infarction: a two-dimensional speckle-tracking study. J Am Soc Echocardiogr.

[40] Ng AC, Bertini M, Borleffs CJ, Delgado V, Boersma E, Piers SR (2010). Predictors of death and occurrence of appropriate implantable defibrillator therapies in patients with ischemic cardiomyopathy. Am J Cardiol.

[41] Buss SJ, Krautz B, Hofmann N, Sander Y, Rust L, Giusca S (2015). Prediction of functional recovery by cardiac magnetic resonance feature tracking imaging in first time ST-elevation myocardial infarction. Comparison to infarct size and transmurality by late gadolinium enhancement. Int J Cardiol.

[42] Khan JN, Nazir SA, Singh A, Shetye A, Lai FY, Peebles C, et al. Relationship of myocardial strain and markers of myocardial injury to predict segmental recovery after acute ST-segment-elevation myocardial infarction. Circ Cardiovasc Imaging. 2016;9(6). 10.1161/CIRCIMAGING.115.003457 27283007

[43] Gaudron P, Kugler L, Hu K, Fraccarollo D, Bauer W, Eilles C (2000). Effect of quinapril initiated during progressive remodeling in asymptomatic patients with healed myocardial infarction. Am J Cardiol.

[44] Zile MR, Baicu CF, Gaasch WH (2004). Diastolic heart failure--abnormalities in active relaxation and passive stiffness of the left ventricle. N Engl J Med.

[45] Mogelvang R, Biering-Sorensen T, Jensen JS (2015). Tissue Doppler echocardiography predicts acute myocardial infarction, heart failure, and cardiovascular death in the general population. Eur Heart J Cardiovasc Imaging.

[46] Shin SH, Hung CL, Uno H, Hassanein AH, Verma A, Bourgoun M (2010). Mechanical dyssynchrony after myocardial infarction in patients with left ventricular dysfunction, heart failure, or both. Circulation..

[47] Schmidt A, Azevedo CF, Cheng A, Gupta SN, Bluemke DA, Foo TK (2007). Infarct tissue heterogeneity by magnetic resonance imaging identifies enhanced cardiac arrhythmia susceptibility in patients with left ventricular dysfunction. Circulation..

[48] Gao P, Yee R, Gula L, Krahn AD, Skanes A, Leong-Sit P (2012). Prediction of arrhythmic events in ischemic and dilated cardiomyopathy patients referred for implantable cardiac defibrillator: evaluation of multiple scar quantification measures for late gadolinium enhancement magnetic resonance imaging. Circ Cardiovasc Imaging..

[49] Klem I, Weinsaft JW, Bahnson TD, Hegland D, Kim HW, Hayes B (2012). Assessment of myocardial scarring improves risk stratification in patients evaluated for cardiac defibrillator implantation. J Am Coll Cardiol.

[50] de Haan S, Meijers TA, Knaapen P, Beek AM, van Rossum AC, Allaart CP (2011). Scar size and characteristics assessed by CMR predict ventricular arrhythmias in ischaemic cardiomyopathy: comparison of previously validated models. Heart..

