# Supplementary material for: Association of cardiovascular magnetic resonance-derived circumferential strain parameters with the risk of ventricular arrhythmia and all-cause mortality in patients with prior myocardial infarction and primary prevention implantable cardioverter defibrillator
Source: J Cardiovasc Magn Reson. 2019 May 16;21:28. doi: 10.1186/s12968-019-0536-5 (PMC6521513; doi:10.1186/s12968-019-0536-5)
Supplement: Supplementary file 1 — Table S1. Unadjusted Cox hazard ratio for the CMR parameters in the acute/subacute phase vs. the chronic stage. (DOCX 19 kb) [file 12968_2019_536_MOESM1_ESM.docx]

### Additional file 1: Table S1. Unadjusted Cox hazard ratio for the CMR parameters in the acute/subacute phase vs. the chronic stage

|  | **Appropriate ICD therapy** | | | | | |
| --- | --- | --- | --- | --- | --- | --- |
|  | **Acute/subacute subgroup**  **(18/72)** | | | **Chronic subgroup**  **(12/49)** | | |
|  | **Cox HR**  **(95%CI)** | ***P* value** | **Harrell’s**  **C-statistic** | **Cox HR**  **(95%CI)** | ***P* value** | **Harrell’s**  **C-statistic** |
| LVEF, per -10% | 1.9 (1.1, 3.4) | 0.022 | 0.67 | 3.1 (1.4, 6.8) | 0.005 | 0.75 |
| Total scar size, per 10 g | 1.1 (0.9, 1.3) | 0.369 | 0.58 | 1.2 (1.0, 1.4) | 0.036 | 0.73 |
| Scar core size, per 10 g | 1.1 (0.8, 1.4) | 0.712 | 0.54 | 1.2 (1.0, 1.4) | 0.069 | 0.67 |
| Scar border size, per 10 g | 1.6 (0.9, 2.6) | 0.093 | 0.59 | 1.6 (1.0, 2.5) | 0.044 | 0.70 |
| Global strain, per +5% | 2.8 (1.3, 5.9) | 0.007 | 0.68 | 3.3 (1.2, 8.7) | 0.017 | 0.69 |
| Peak systolic strain rate, per +0.25 1/s | 2.1 (1.2, 3.7) | 0.011 | 0.68 | 3.1 (1.3, 7.6) | 0.014 | 0.70 |
| *Extent of impaired strain, %* |  |  |  |  |  |  |
| Severely (<-5%) | 1.6 (1.0, 2.3) | 0.033 | 0.63 | 1.5 (1.0, 2.2) | 0.051 | 0.64 |
| Moderately (-5, -10%) | 1.9 (1.3, 2.7) | 0.002 | 0.72 | 1.8 (1.1, 3.1) | 0.025 | 0.69 |
| Mildly (-10, -15%) | 0.8 (0.4, 1.5) | 0.476 | 0.56 | 0.6 (0.3, 1.4) | 0.259 | 0.62 |
| Early diastolic strain rate, per -0.25 1/s | 1.1 (1.0, 1.1) | 0.137 | 0.59 | 1.3 (1.1, 1.5) | 0.001 | 0.79 |
| Late diastolic strain rate, per -0.25 1/s | 1.1 (1.0, 1.2) | 0.052 | 0.64 | 1.1 (1.0, 1.3) | 0.076 | 0.66 |
| Mechanical dispersion, per +25 ms | 1.5 (0.9, 2.3) | 0.113 | 0.59 | 0.8 (0.5, 1.6) | 0.601 | 0.50 |
| Sphericity index, per +0.1 | 1.0 (0.7, 1.6) | 0.843 | 0.51 | 1.3 (0.9, 1.8) | 0.218 | 0.55 |

LV: left ventricle. LVEF: left ventricular ejection fraction. Extent of impaired strain: percentage of LV segments with strain >-15%. Acute/subacute subgroup: CMR <40 days post myocardial infarction or <3 months following revascularization. Chronic subgroup: CMR >40 days post myocardial infarction or >3 months following revascularization
